# Supplementary material for: Scater: pre-processing, quality control, normalization and visualization of single-cell RNA-seq data in R
Source: Bioinformatics. 2017 Jan 14;33(8):1179–86. doi: 10.1093/bioinformatics/btw777 (PMC5408845; doi:10.1093/bioinformatics/btw777)
Supplement: Supplementary Data [file btw777_supp.zip › case_study.html]

SCATER CASE STUDY


Code 

- Show All Code
- Hide All Code

# SCATER CASE STUDY

#### *Davis McCarthy, Kieran Campbell, Aaron Lun & Quin Wills*

#### *2016-09-07*

1. **OVERVIEW:** Brief introduction to scater and the case study data.
2. **GETTING STARTED:** Import data/meta-data, and run automated QC.
3. **QC THE CELLS:** Exploratory plots and quality control of the cells.
4. **QC THE GENES:** Exploratory plots and quality control of the genes.
5. **NORMALISE THE DATA**: Unwanted technical effects in the data, and corrective normalisation.
6. **DOWNSTREAM ANALYSIS:** An example of further analysis.
7. **SESSION INFORMATION:** Information for bioinformaticians, such as the R session used to run this case study.

---

## 1. OVERVIEW

This case study is designed to walk you through key scater features from importing and easily QC’ing your single-cell data, through to seamless integration with other data types and Bioconductor packages. If you’re new to single cell RNA-seq, this case study should help you get started with your own data, irrespective of the platforms used to process the cells for sequencing. As scater is an R package, some of the basic work flow is also implemented as a shiny GUI for those less familiar with R coding. The aim is to get you from sequencer to higher level analysis and modelling with minimal fuss.

The RNA-seq data provided here are 73 cells from two lymphoblast cell lines of two unrelated individuals. Cells were captured, lysed, and cDNA generated using a popular microfluidics platform. The processing of the two cell lines was replicated across two machines, with the nuclei of the two cell lines stained with different dyes before mixing on each machine. Cells were imaged before lysis, with an example image provided together with this data.

To run this case study you will require the following packages in addition to scater: EBImage, qvalue, and mclust.

---

## 2. GETTING STARTED

Scater processing can begin with sequencer FASTQ files, using the inbuilt Kallisto pseudo-aligner. This is dicussed here and in the supporting help files. Here we assume that read counts have already been provided from a more traditional RNA-seq pipeline, and read them in as follows:

```
reads <- as.matrix( read.csv( "reads.csv", row.names = 1 ) )
```

In keeping with best practice in object-oriented bioinformatics, *scater* not only allows you to store your data in a standardised format, but also to associate it with experimental and bioinformatic meta-data. Experimental meta-data are any information associated with individual cells (also called ‘samples’), from sequencing metrics to experimental batching and other experimental cell phenotypes. The bioinformatic meta-data are any information related to the genes (also called ‘features’). These can be read in, combined with the sequencing data, and extracted as shown below. For this example we’ll use the automated log-scaled counts per million generated by *scater*, but see `calculateTPM()` as an example function for generating alternative RNA-seq read count formats.

View the first few entries for the cell metadata:

```
cells <- AnnotatedDataFrame( read.csv( "cells.csv", row.names = 1 ) ) # row names match column names in reads
genes <- AnnotatedDataFrame( read.csv( "genes.csv", row.names = 1 ) ) # row names match row names in reads
scData <- newSCESet( countData = reads, phenoData = cells, featureData = genes, lowerDetectionLimit = 3 ) 
head(pData(scData))  # view some sample meta-data
```

```
##                    sample_type c1_machine c1_capture_site
## cellA_1_01 cell from patient A          1               1
## cellA_1_05 cell from patient A          1               5
## cellA_1_13 cell from patient A          1              13
## cellA_1_19 cell from patient A          1              19
## cellA_1_36 cell from patient A          1              36
## cellA_1_49 cell from patient A          1              49
##            cdna_recovered_in_ng_per_ul
## cellA_1_01                       1.710
## cellA_1_05                       1.920
## cellA_1_13                       0.972
## cellA_1_19                       1.180
## cellA_1_36                       3.380
## cellA_1_49                       0.988
```

View the first few entries for the feature metadata:

```
fData( scData )[1:3, 1:2] # view some feature meta-data
```

```
##                 hgnc_symbol entrez_gene_id
## ENSG00000000003      TSPAN6           7105
## ENSG00000000419        DPM1           8813
## ENSG00000000457       SCYL3          57147
```

Scater comes with an inbuilt QC function, which is worth running once all data has been imported as an SCESet object. To do this, first define any feature (gene) and sample (cell) controls. Here we define ERCC synthetic RNA spike-ins as our feature controls, with bulk sequencing samples and empty capture sites (i.e. no cells) as blank controls.

```
spikes <- grepl( "ERCC-", row.names( scData ) ) # ERCC spike-ins
blanks <- scData$sample_type == "blank (empty site) control" # negative/background controls
bulks <- grepl( "bulk control", scData$sample_type ) # samples of several hundred cells
scData <- calculateQCMetrics(
    scData, feature_controls = list( spikes = spikes ), 
    cell_controls = list( bulks = bulks, blanks = blanks ) )
```

---

## 3. QC THE CELLS

A useful first step is flagging/failing poorly performing cells. This can be done from the sample meta-data using the automated QC metrics generated above, any additional sequencing metrics from sequencing aligner/mapping software, and additional cell phenotypes such as from imaging. For the sake of demonstration, here we focus on four metrics. Others you may want to consider are % reads mapped to mitochondrial genes, library PCR duplication rate, and mean sequencing bias per cell.

```
scData$use <- (scData$cdna_recovered_in_ng_per_ul > 0.5 & #sufficient cDNA per cell 
                   scData$pct_counts_feature_controls < 1 & # sufficient endogenous RNA
                   !scData$total_counts < 1e5 & # sufficient reads mapped to features
                   !scData$filter_on_total_features & # remove cells with unusual numbers of genes
                   !scData$is_cell_control # controls shouldn't be used in downstream analysis
)
```

Box plots aren’t particularly useful for visualising sparse data, so `plot()` applied to an SCESet object helps visualise all cells as a cumulative proportion of reads per cell. You can see from the plot below that the two failed cells have curves that look more like the blank controls.

```
plot( scData, block1 = "sample_type", block2 = "c1_machine", colour_by = "use",
      exprs_values = "counts")
```

Scater allows users total flexibility to run their favourite dimension reduction methods, as decribed here and in the supporting help files. Here we use `plotPCA()` to further explore the cells. You can see that the two cell lines cluster separately, with the respective bulk control samples occuring within the correct clusters. With all single-cell work there is background RNA (secreted or from lysed cells), with the blanks here clustering between the two samples. One feature to look out for is if individual blanks cluster strongly with one or other cell type, suggesting that the background admixture isn’t homogeneous. As there are more samples here from patient B, blanks demonstrate a stronger patient B admixture.

```
plotPCA( scData, colour_by = "use", shape_by = "sample_type" )
```

```
plotPCA( scData[, grep("cell", scData$sample_type)], colour_by = "use", 
         shape_by = "sample_type")
```

  Another option available in *scater* is to conduct PCA on a set of QC metrics. The advantage of doing this is that the QC metrics focus on technical aspects of the libraries that are likely to distinguish problematics cells. Automatic outlier detection on PCA plots using QC metrics is available to help identify potentially problematic cells.

By default, the following metrics are used for PCA-based outlier detection:

- `pct_counts_top_100_features`
- `total_features`
- `pct_counts_feature_controls`
- `n_detected_feature_controls`
- `log10_counts_endogenous_features`
- `log10_counts_feature_controls`

A particular set of variables to be used can be specified with the `selected_variables` argument as shown in the example below.

```
## PCA on the phenoData cannot handle missing values
## for the exercise here we thus set cdna_recovered_in_ng_per_ul to 100 where 
## there are NA values actually (creating a new dummy variable)
cdna_temp <- scData$cdna_recovered_in_ng_per_ul
cdna_temp[is.na(cdna_temp)] <- 100
scData$cdna_recovered_in_ng_per_ul_no_miss <- cdna_temp
scData <- plotPCA( scData, size_by = "use", shape_by = "sample_type", 
                   pca_data_input = "pdata", detect_outliers = TRUE,
                   return_SCESet = TRUE, 
                   selected_variables = c("cdna_recovered_in_ng_per_ul_no_miss",
                                          "pct_counts_top_100_features",
                                          "total_features", 
                                          "pct_counts_feature_controls",
                                          "n_detected_feature_controls", 
                                          "log10_counts_endogenous_features",
                                          "log10_counts_feature_controls"))
```

```
## The following cells/samples are detected as outliers:
## cellA_1_56
## cellB_1_38
## cellB_2_34
## cellB_2_35
## blank_1_03
## blank_1_20
## blank_1_94
## blank_2_02
## blank_2_14
## blank_2_51
## bulkA
## bulkB
## Variables with highest loadings for PC1 and PC2:
## 
##                                               PC1          PC2
## ------------------------------------  -----------  -----------
## pct_counts_feature_controls             0.4525993    0.1308720
## pct_counts_top_100_features             0.4338506    0.0155969
## cdna_recovered_in_ng_per_ul_no_miss     0.3823536    0.4373666
## log10_counts_feature_controls           0.3165694   -0.5613388
## n_detected_feature_controls             0.0930809   -0.6417230
## total_features                         -0.3829124    0.1892048
## log10_counts_endogenous_features       -0.4530244   -0.1692090
```

```
plotPCA( scData[, grep("cell", scData$sample_type)], colour_by = "outlier", 
         shape_by = "use", pca_data_input = "pdata")
```

```
plotDiffusionMap( scData[, grep("cell", scData$sample_type)], 
                  colour_by = "outlier", shape_by = "use", 
                  pca_data_input = "pdata")
```

Having detected potential outliers on the phenotype data (i.e. cell metrics) we can see where these cells appear on PCA and diffusion map plots based on the expression data.

```
plotPCA( scData[, grep("cell", scData$sample_type)], ncomponents = 3,
         shape_by = "use", pca_data_input = "pdata", colour_by = "outlier")
```

```
p1 <- plotPCA( scData, colour_by = "use", shape_by = "sample_type", 
         size_by = "outlier" ) + ggtitle("PCA")
p2 <- plotDiffusionMap( scData, colour_by = "use", shape_by = "sample_type", 
         size_by = "outlier" ) + ggtitle("Diffusion map")
multiplot(p1, p2, cols = 2)
```

With *scater*, any specific set of features based on prior knowledge can be used for PCA, t-SNE or diffusion maps. A feature set to use can be defined by supplying the `feature_set` argument to `plotPCA`, `plotTSNE` or `plotDiffusionMap`. This allows, for example, using only housekeeping features or control features or cell cycle genes to produce reduced-dimension plots. The plots below use only the spike-in genes defined as such earlier.

```
p1 <- plotPCA( scData, feature_set = fData(scData)$is_feature_control, 
               colour_by = "use", shape_by = "sample_type", 
               size_by = "outlier" ) + ggtitle("PCA")
p2 <- plotDiffusionMap( scData, feature_set = fData(scData)$is_feature_control, 
                        colour_by = "use", shape_by = "sample_type", 
                        size_by = "outlier" ) + ggtitle("Diffusion map")
multiplot(p1, p2, cols = 2)
```

The following plot uses only the genes annotated as cell cycle genes (this annotation was included in the genes information loaded into the SCESet object at the start). Points are coloured by expression of RAN (ENSG00000132341), a gene associated with the G2/M phase of the cell cycle.

```
cell_cycle_genes <- !is.na(fData(scData)$cell_cycle_phase)
sum(cell_cycle_genes)
```

```
## [1] 488
```

```
most_exprs_ccycle <- which(cell_cycle_genes & 
                               fData(scData)$total_feature_counts > 100000)
p1 <- plotPCA(scData, feature_set = cell_cycle_genes, 
               colour_by = featureNames(scData)[most_exprs_ccycle[8]], 
               shape_by = "sample_type", 
               size_by = "use" ) + ggtitle("PCA")
p2 <- plotDiffusionMap( scData, feature_set = cell_cycle_genes, 
                        colour_by = featureNames(scData)[most_exprs_ccycle[8]], 
                        shape_by = "sample_type", 
                        size_by = "use" ) + ggtitle("Diffusion map")
multiplot(p1, p2, cols = 2)
```

`plotPhenoData()` can be used to explore specific sample meta-data values. For example, in the plot below we can see strong association between cDNA recovery and number of genes detected in a cell. The two failed cells demonstrate low gene coverage (total features) and cDNA recovery.

```
plotPhenoData( scData, aesth = aes_string( x = "cdna_recovered_in_ng_per_ul", 
                                         y = "total_features", colour = "use" ) )
```

One element of QC that can prove challenging to judge from the above criteria is if more than one cell ( or cell debris from other cells ) is being sequenced. With most cell processing platforms a small percentage of cells won’t be truly single. One solution is imaging individual cells, which also allows for the incorporation of fluorescence phenotypes such as viability and nuclear content ( for cell cycle analysis ). Using the EBImage package, an example image of a stained cell is processed and displayed below, with results incorporated into the sample meta-data.

```
imageQC <- function(cell) { 
    require( "EBImage" )
    pass <- TRUE # if the cell should be passed or failed
    
    im <- readImage( paste( cell, ".tif", sep = "" ) ) # read in image TIFF for cell of interest
    im <- thresh( im, w = 20, h = 20, offset = 0.1 ) # convert to binary fluorescence matrix 
    display( im, method = "raster" ) # view fluorescent spot(s)
    im <- bwlabel(  opening(  im,  makeBrush( 3, "gaussian" ) ) ) # label clusters of glowing spots
    
    cl <- sort( table( im )[-1], decreasing = TRUE ) # pixels per fluorescent spot
    if ( sum( cl > 1e2 ) != 1 ) pass <- FALSE # pass if a single spot greater than 100 pixels 
    
    sh <- prcomp( which( im == names( cl )[1],  arr.ind = TRUE ) )  # a non-circular shape may be a doublet/dividing cell
    if ( sh$sdev[1] ^ 2/sum( sh$sdev ^ 2 ) > 0.75 ) pass <- FALSE
    
    pass
}

pData( scData )["cellB_1_04", "use"] <- 
    (pData( scData )["cellB_1_04", "use"] & imageQC( "cellB_1_04" ) )
```

---

## 4. QC THE GENES

Scater allows you to set minimum QC thresholds for a gene to be considered sufficiently expressed in your downstream analysis. Here, using the inbuilt `is_exprs()` function, we enforce that a gene must have least three reads in at least three cells in both replicates.

```
reps <- scData$c1_machine # the machine replicates
is_exprs(scData) <- counts(scData) >= 3
exprsThresh <- sapply( 1:2,  
               function(r) apply( is_exprs( scData )[, scData$use & reps == r], 1, 
                                    function(x) sum( x ) >= 3 ) )
fData( scData )$use <- (apply( exprsThresh, 1, all ) & # endogenous genes above the threshold are used downstream
                            !fData( scData )$is_feature_control_spikes)
```

It can be useful to plot gene expression frequency versus mean expression level to assess the effects of technical dropout in the dataset. We fit a non-linear least squares curve for the relationship between expression frequency and mean expression and use this to define the number of genes above high technical dropout and the numbers of genes that are expressed (here defined as at least 4 counts) in at least 50% and at least 25% of cells. A subset of genes to be treated as feature controls can be specified, otherwise any feature controls previously defined are used.

```
plotQC(scData, type = "exprs")
```

It can also be useful to look at total expression levels across gene biotypes. The plot below shows the distributions of log-10 scale total gene counts for genes of different annotated biotypes. The biotypes are ordered by median total counts, and the jittered points beneath the violin plots indiciate the number of genes in the biotype. We want to see many protein-coding genes with reasonably high expression. This is exactly what we see here, with protein-coding genes appearing as the biotype with second-highest median expression behind the very small (but highly expressed) category of Mt\_rRNA genes.

```
oo <- order(unlist(dplyr::summarise(dplyr::group_by(fData(scData), gene_biotype),
                 median(log10_total_feature_counts))[,2]), decreasing = TRUE)
fData(scData)$biotype <- factor(
    fData(scData)$gene_biotype, 
    levels = levels(as.factor(fData(scData)$gene_biotype))[oo])

plotFeatureData(scData, aes(x = biotype, y = log10_total_feature_counts),
                theme_size = 6) +
    theme(axis.text.x = element_text(angle = 90, hjust = 0, vjust = 0.5))
```

The `plotQC()` function provides several useful QC plots, such as the example below that considers the the number of reads consumed by the top 50 expressed genes. Aside from spike-ins, these are typically mitochondrial and housekeeping genes. Here, as with most single-cell experiments, a large proportion of reads are being are taken up by uninteresting biology.

To make gene labels more interpretable we append the gene symbol to the Ensembl gene IDs.

```
fData(scData)$gene_symbol <- fData(scData)$hgnc_symbol
fData(scData)$gene_symbol[is.na(fData(scData)$gene_symbol)] <-
    featureNames(scData)[is.na(fData(scData)$gene_symbol)]
featureNames(scData) <- paste(fData(scData)$hgnc_symbol, featureNames(scData),
                              sep = "_")
```

```
plotQC( scData[fData( scData )$use, pData( scData )$use], 
        type = "highest-expression", 
        col_by_variable = "sample_type", feature_names_to_plot = "gene_symbol")
```

---

## 5. NORMALISE THE DATA

Once you have filtered cells and genes, a next step is to explore technical drivers of variability in the data to inform data normalisation before downstream analysis.

Experimental design is a critical, but neglected, aspect of studies. To the best of our knowledge, methods like those described in this section for exploring experimental and QC variables and the experimental design, do not feature in any scRNA-seq software packages apart from *scater*. There are a very large number of potential confounders, artifacts and biases in studies. Exploring the effects of such explanatory variables (both those recorded during the experiment and computed QC metrics) is crucial for appropriate modeling of the data. The  package provides a set of methods specifically for quality control of experimental and explanatory variables, which will be demonstrated briefly here.

### Finding important explanatory variables

Using the `plotPCA()` function we can see that principal component one is driven by differences between the two machine replicates, which in turn is due to differences in gene coverage. Differences in number of detected genes is a common driver of cell clustering and can be result of biology (e.g. different cell types, cell cycle). However, it often has a strong technical component due to variably recovered RNA, reverse transcription, or library amplification. Its effect can also be notably non-linear, affecting low expressed and high expressed genes differently. The `plotQC()` function can be used to explore the the marginal % variance explained (per gene) of the various technical factors. In the second plot we can see that it’s not unusual for gene coverage to explain more than 10% of the expression variance of a gene.

```
plotPCA( scData[fData( scData )$use, scData$use],  # only plot genes and cells of interest
         colour_by = "sample_type", 
         shape_by = "c1_machine", 
         size_by = "total_features" )
```

```
plotDiffusionMap( scData[fData( scData )$use, scData$use], ntop = 5000,
                  colour_by = "sample_type", 
                  shape_by = "c1_machine", 
                  size_by = "total_features" )
```

The relative importance of different explanatory variables can be explored with some of the `plotQC` function options. Supplying the `type = "expl"` argument to `plotQC` computes the marginal \(R^2\) for each variable in the when fitting a linear model regressing expression values for each gene against just that variable, and displays a density plot of the gene-wise marginal \(R^2\) values for the variables. The default approach looks at all variables in the slot of the object and plots the top `nvars_to_plot` variables (default is 10).

Alternatively, one can choose a subset of variables to plot in this manner, which we do here. The density curves for marginal \(R^2\) show the relative importance of different variables for explaining variance in expression between cells.

```
plotQC( scData[fData( scData )$use, scData$use], 
        type = "explanatory-variables", 
        variables = c("pct_counts_top_100_features", "total_features", 
                      "pct_counts_feature_controls", "c1_machine",
                      "n_detected_feature_controls", 
                      "log10_counts_endogenous_features",
                      "log10_counts_feature_controls", "sample_type") )
```

This analysis indicates that total number of features detected and the sequencing depth (number of counts) for endogenous genes, in particular, have substantial explanatory power for many genes, so these variables are good candidates for conditioning out in a normalisation step, or including in downstream statistical models. The number of detected feature controls (spike-in genes) does not appear to be an important explanatory variable.

One can also easily produce plots to identify principal components that correlate with experimental and QC variables of interest. The function `plotQC` with the option `type = "find-pcs"` ranks the principal components in decreasing order of \(R^2\) from a linear model regressing PC value against the variable of interest. The default behaviour is to show the relationships between the variable of interest and the six principal components with the strongest relationship to the variable (as measured by \(R^2\)). This works both for continous and categorical variables. This type of plot can indicate which explanatory variables may be driving differences between cells as detected by PCA and highlight which PCs are associated with the variable. The “total features” variable shows very strong correlation with both principal components 1 and 2.

```
p1 <- plotQC( scData[fData( scData )$use, scData$use], 
        type = "find-pcs", variable = "total_features" )
p2 <- plotQC( scData[fData( scData )$use, scData$use], 
        type = "find-pcs", variable = "sample_type" )
multiplot(p1, p2, cols = 2)
```

The cells in this dataset were processed on two C1 machines, which gives rise to a substantial batch effect. The QC plots below show that the C1 machine factor is correlated with the first principal component. This effect is likely caused by the difference in amount of cDNA recovered per cell, which is even more strongly correlated with the first principal component. Thus, differences in batches are a major source of variation between the cells.

```
scData$c1_machine <- as.factor(scData$c1_machine)
p1 <- plotQC( scData[fData( scData )$use, scData$use], 
        type = "find-pcs", variable = "c1_machine" )
p2 <- plotQC( scData[fData( scData )$use, scData$use], 
        type = "find-pcs", variable = "cdna_recovered_in_ng_per_ul")
multiplot(p1, p2, cols = 2)
```

```
## `stat_bindot()` using `bins = 30`. Pick better value with `binwidth`.
```

The `plotQC()` function can also be used to produce a pairs plot of explanatory variables (with the same calls as above, but with `method = "pairs"`). The plot below shows this use case for looking at the % counts from the top 100 most-expressed genes, the total number of expressed genes, the % of counts from feature controls, the number of detected feature controls, the number of counts (on the log-10 scale) from endogenous features, the number of counts (log-10 scale) from feature controls and sample type. The explanatory variables are ordered by the median \(R^2\) of the variable across all genes, and this value is reported on the plot. This type of plot is useful for finding correlations between experimental and QC variables with substantial explanatory power.

```
plotQC( scData[fData( scData )$use, scData$use], 
        type = "expl", method = "pairs", 
        variables = c("pct_counts_top_100_features", "total_features", 
                      "pct_counts_feature_controls", "c1_machine",
                      "n_detected_feature_controls", 
                      "log10_counts_endogenous_features",
                      "log10_counts_feature_controls", "sample_type"),
        theme_size = 6)
```

After important explanatory variables have been identified with the tools shown above, their effects can be accounted for in subsequent statistical models, or they can be conditioned out using `normaliseExprs()`, if so desired. If a design matrix incorporating a selection of explanatory variables is supplied as an argument to `normaliseExprs`, then normalised expression values returned for each feature will be the residuals from a linear model fitted with the design matrix, after any size-factor normalisation has been applied to the expression data.

Normalising single-cell RNA-seq data is a topic in its infancy, but many of the basic principles still apply. How much you choose to initially correct for technical factors depends on your question of interest and the ease with which they can be accounted for in downstream models.

### Scaling normalisation

In *scater* it is easy to perform scaling normalisation using the “TMM” approach (the default in the edgeR package), the “RLE” approach (default in the DESeq package), an “upper-quartile approach” (as proposed by Bullard et al (2010)) or simple scaling by total counts. Size-factor normalisation can be carried out on a subsetted SCESet object, normalising either to ERCC spike-in genes or to endogenous genes if desired. Under certain circumstances either may be appropriate. Careful thought should be given to applying scale-factor normalisation as underlying assumptions from methods developed for bulk RNA-seq may not be appropriate for all single-cell datasets.

However, Lun et al (2016) recently published a size-factor normalisation method specifically designed for scRNA-seq data. This method performs much better on single-cell data and its implementation in the Bioconductor package *scran* allows seamless integration into the *scater* workflow. (The *scran* package itself depends on *scater*).

We recommend using the *scran* size-factor normalisation approach and demonstrate its use here.

In the code below we use the `filter` function to select a subset of cells from an `SCESet` object (this method works just as the `filter` function in the `dplyr` package on which it is based).

```
## subset to form a QC's version of the data
scDataQC <- filter(scData, use)
scDataQC <- scDataQC[fData(scData)$use | fData(scData)$is_feature_control,]
ercc_genes <- fData(scDataQC)$is_feature_control
endog_genes <- !ercc_genes

## size factor normalisation with scran 
qclust <- quickCluster(scDataQC, min.size = 20)
scDataQC <- computeSumFactors(scDataQC, sizes = 10, clusters = qclust)
summary(scDataQC$size_factor)
```

```
##    Min. 1st Qu.  Median    Mean 3rd Qu.    Max. 
##  0.1611  0.7464  0.9806  1.0000  1.1980  2.8450
```

```
scDataQC <- normalise(scDataQC)

isSpike(scDataQC) <- "spikes"
scDataQC_spikenorm <- computeSpikeFactors(scDataQC)
summary(scDataQC_spikenorm$size_factor)
```

```
##     Min.  1st Qu.   Median     Mean  3rd Qu.     Max. 
## 0.006421 0.448600 0.796500 1.000000 1.322000 6.217000
```

```
scDataQC_spikenorm <- normalise(scDataQC_spikenorm)

set_exprs(scDataQC, "ercc_norm_exprs") <- exprs(scDataQC_spikenorm)
set_exprs(scDataQC, "endog_norm_exprs") <- exprs(scDataQC)
## subset again so that only endogenous genes are used
scDataQC <- scDataQC[fData(scDataQC)$use,]
plt_pca_ercc_norm <- plotPCA(
    scDataQC, exprs_values = "ercc_norm_exprs", size_by = "total_features",  
    colour_by = "sample_type", shape_by = "c1_machine") + 
    ggtitle("PCA - ERCC size-factor normalisation") + 
    theme(legend.position = "bottom")
plt_pca_endog_norm <- plotPCA(
    scDataQC, exprs_values = "endog_norm_exprs", size_by = "total_features",  
    colour_by = "sample_type", shape_by = "c1_machine") + 
    ggtitle("PCA - endogenous size-factor normalisation") + 
    theme(legend.position = "bottom")
multiplot(plt_pca_ercc_norm, plt_pca_endog_norm, cols = 2)
```

In the PCA plots above we see that the first principal component seems to separate cells based on the C1 machine used to process them, even after appropriate size-factor normalisation. The `plotQC` function confirms this in the plot below: principal component 1 is the PC most highly correlated with C1 machine.

We would prefer such technical effects to be removed by normalisation, so below we demonstrate a further alternative approach. We see how “customised” normalisation approaches can be easily incorporated into the *scater* workflow.

### Expression-conditional normalisation

In this example we normalise (by cell) and standardise (by gene) counts conditioned on expression level, showing the PCA for the corrected data. One advantage of this approach is that a biologically ‘noisy’ gene is naturally defined as one with greater dispersion than other genes at a similar expression level. In the normalised data these are genes with a mean absolute deviation greater than 1.

```
rp <- scData$c1_machine[scData$use] # replicates
mn <- sapply( 1:2,  # mean log expression per replicate
              function(r) rowMeans(exprs( scData )[fData( scData )$use, 
                                                   scData$use][, rp == r])) 

exprsNorm <- counts( scData )[fData( scData )$use, pData( scData )$use] # read counts to normalise
wn <- floor( 0.05*nrow( scData ) ) # consider a sliding window of 10% of genes
for (r in 1:2) { # normalise per replicate
  exprsNorm[, rp == r] <- t( sapply( row.names( exprsNorm ), function(g){
    nh <- head( sort( abs( mn[, r] - mn[g, r] ) ), wn ) # neighbourhood of similarly expressing genes
    nh <- exprsNorm[names( nh ), rp == r]
    nh <- log2( t( t( nh ) / colSums( nh ) ) * 1e5 + 1 ) # locally normalise
    nh <- nh - mean( nh ) # locally standardise 
    nh <- nh / mean( abs( nh ) )
    nh[1, ]
  } ) )  
}

### add normalised expression values to SCESet object and make PCA plot
norm_exprs(scDataQC) <- exprsNorm
plt_cond_norm <- plotPCA(
    scDataQC, exprs_values = "norm_exprs", shape_by = "c1_machine", 
        colour_by = "sample_type", size_by = "total_features", 
        scale_features = FALSE) + 
    ggtitle("PCA - expression conditional normalisation") + 
    theme(legend.position = "bottom")
plt_cond_norm
```

After this normalisation procedure we see that the first principal component now separates cells from the two patients and neither the first nor second principal component appears influenced by the C1 machine used to process the cells.

Furthermore, C1 machine shows no correlation with the top principal components, as shown in the plot below.

### Regressing out known sources of variation

Another option is to regress out effects of technical factors in the normalisation step.

```
design <- model.matrix(~scDataQC$log10_counts_endogenous_features +
                           scDataQC$pct_counts_top_100_features +
                           scDataQC$pct_counts_feature_controls +
                           scDataQC$c1_machine)
set_exprs(scDataQC, "norm_exprs_resid") <- norm_exprs(
    normaliseExprs(scDataQC, design = design,
                   method = "none", exprs_values = "exprs",
                   return_norm_as_exprs = FALSE) )

plt_sf_norm_resid <- plotPCA(
    scDataQC, exprs_values = "norm_exprs_resid", shape_by = "c1_machine", 
        colour_by = "sample_type", size_by = "total_features", 
        scale_features = TRUE) + 
    ggtitle("PCA - size-factor normalisation residuals") + 
    theme(legend.position = "bottom")
plt_sf_norm_resid
```

As we see here, this simple regression approach is here also able to substantially remove the C1 machine effect from that the data so that the first principal component now separates cells by patient.

We can also check the results that would be obtained when regressing out technical effects computed from the expression values but *not* C1 machine. This can give us an idea of what effects could be removed if we did not know that cells were process on different C1 machines and only had access to the QC metrics we could compute from the expression data, or other sample metadata information provided.

The PCA plots below show the results after regressing out log10(counts) from endogenous features, % counts from the top 100 most-expressed features, the percentage counts from control features and the log10(counts) from feature controls (top) and after regressing out all of those effects plus cDNA recovered from each cell in ng per microlitre.

```
design2 <- model.matrix(~scDataQC$log10_counts_endogenous_features +
                            scDataQC$pct_counts_top_100_features +
                            scDataQC$pct_counts_feature_controls +
                            scDataQC$log10_counts_feature_controls)
set_exprs(scDataQC, "norm_exprs_resid2") <- norm_exprs(
    normaliseExprs(scDataQC, design = design2,
                   method = "none", exprs_values = "exprs",
                   return_norm_as_exprs = FALSE) )

plt_cond_norm_resid2 <- plotPCA(
    scDataQC, exprs_values = "norm_exprs_resid2", shape_by = "c1_machine", 
        colour_by = "sample_type", size_by = "total_features", 
        scale_features = FALSE) + 
    ggtitle("PCA - size-factor normalisation residuals technical factors") + 
    theme(legend.position = "bottom")

design3 <- model.matrix(~scDataQC$log10_counts_endogenous_features +
                            scDataQC$pct_counts_top_100_features +
                            scDataQC$pct_counts_feature_controls +
                            scDataQC$log10_counts_feature_controls +
                            scDataQC$cdna_recovered_in_ng_per_ul_no_miss)
set_exprs(scDataQC, "norm_exprs_resid3") <- norm_exprs(
    normaliseExprs(scDataQC, design = design3,
                   method = "none", exprs_values = "exprs",
                   return_norm_as_exprs = FALSE) )

plt_cond_norm_resid3 <- plotPCA(
    scDataQC, exprs_values = "norm_exprs_resid3", shape_by = "c1_machine", 
        colour_by = "sample_type", size_by = "total_features", 
        scale_features = FALSE) + 
    ggtitle("PCA - size-factor normalisation residuals incl cDNA") + 
    theme(legend.position = "bottom")

multiplot(plt_cond_norm_resid2, plt_cond_norm_resid3)
```

Here, regressing out only the technical QC metrics does not remove the major effect of C1 machine, but regressing out cDNA concentration as well does (so the first principal component now separates the two patients). Thus, for removing known effects, the regression approach implemented in *scater* is effective, but more needs to be done to find *hidden* effects such as unknown batch effects, or similar.

### Normalisation with `removeBatchEffect` from `limma`

Look at the performance of `removeBatch` from the `limma` package:

```
design_rbe <- model.matrix(~as.character(scDataQC$sample_type))
rbe_norm_exprs <- removeBatchEffect(exprs(scDataQC), batch = scDataQC$c1_machine,
                                    design = design_rbe)
rbe_norm_exprs <- removeBatchEffect(get_exprs(scDataQC, "endog_norm_exprs"), 
                                    batch = scDataQC$c1_machine,
                                    design = design_rbe)
set_exprs(scDataQC, "norm_exprs_rbe") <- rbe_norm_exprs
plt_rbe_norm <- plotPCA(
    scDataQC, exprs_values = "norm_exprs_rbe", shape_by = "c1_machine", 
        colour_by = "sample_type", size_by = "total_features") + 
    ggtitle("PCA - removeBatchEffect normalisation") + 
    theme(legend.position = "bottom")
plt_rbe_norm
```

After normalisation with `removeBatchEffect`, the patient effect is still not the strongest one picked out by PCA. Nevertheless, there is now no correlation between PCs and C1 machine, as the plot below shows.

```
plotQC(scDataQC, type = "find-pcs", variable = "c1_machine", 
       exprs_values = "norm_exprs_rbe")
```

```
## `stat_bindot()` using `bins = 30`. Pick better value with `binwidth`.
```

### Batch correction with `RUVSeq`

Look at the performance of the RUVs method:

```
# One approach to finding and removing hidden, unwanted sources of variability is
# the `RUVSeq` package.

scDataRUV <- filter(scData, use)
scDataRUV <- scDataRUV[fData(scData)$use | fData(scData)$is_feature_control,]
scIdx <- matrix(-1, ncol = max(table(scDataRUV$sample_type)), nrow = 2)
scIdx[1,] <- which(scDataRUV$sample_type == "cell from patient B")
tmp <- which(scDataRUV$sample_type == "cell from patient A")
scIdx[2, 1:length(tmp)] <- tmp
# cIdx <- (fData(scDataRUV)$is_feature_control & fData(scDataRUV)$n_cells_exprs >= 10) 
## Running RUVs
cIdx <- rownames(scDataRUV)
ruv_list <- list()
for (k in 1:4) {
    ruvs <- RUVs(counts(scDataRUV), cIdx, k = k,
                 scIdx = scIdx, isLog = FALSE)
    norm_counts(scDataRUV) <- ruvs$normalizedCounts
    exprs(scDataRUV) <- edgeR::cpm.default(norm_counts(scDataRUV), log = TRUE, 
                                           prior.count = scDataRUV@logExprsOffset)
    plt_norm_ruv <- plotPCA(
        scDataRUV, exprs_values = "exprs", shape_by = "c1_machine", 
        colour_by = "sample_type", size_by = "total_features", 
        scale_features = TRUE) + 
        ggtitle(paste("PCA - RUVs normalisation: k =", k)) + 
        theme_bw(8) + theme(legend.position = "bottom")
    ruv_list[[k]] <- plt_norm_ruv
}

figplot_ruv <- cowplot::plot_grid(plotlist = ruv_list, labels = letters, ncol = 2)
figplot_ruv
```

```
cowplot::save_plot("../figures/supplement/ruvs_figure.png", figplot_ruv, 
                   ncol = 3, nrow = 3, base_height = 5, base_aspect_ratio = 0.9)
```

Just removing the first latent variable found with RUVs now effectively splits cells from the two patients in the first principal component of the adjusted expression values.

The QC plot below confirms that the first principal component is now highly correlated with patient.

```
plotQC(scDataRUV, type = "find-pcs", variable = "sample_type")
```

Thus, after convenient pre-processing, QC and normalisation with , the data are well organised (with feature and cell metadata and many data transformations), clean and tidy, and are ready for further statistical modeling and analysis.

---

## 6. DOWNSTREAM ANALYSIS

Expression data at a single-cell resolution not only allows testing for differential expression, but exploring how this is dependent on sub-types of cells and/or how genes coexpress with each other across cells. As with normalisation, single-cell analysis methodology is an area in its infancy and deserving of discussion that is beyond this case study. Here we simply demonstrate how QC’ed and normalised data contained within an SCESet object allows for easy downstream interrogation. Let’s assume we are interested in defining differential expression as change in expression frequency. This can be done with a standard generalised linear model and the `qvalue` package to control false discovery rates.

```
library( "qvalue" )

sm <- scDataQC$sample_type # the two samples
de <- data.frame( t( apply( norm_exprs(scDataQC) > 0, 1, # test change in expression frequency 
                            function( y ) coef(summary(glm(y ~ sm, family = "binomial" ) ) )[2, c(1,4)])),  check.names = FALSE)
de$qvalue <- qvalue( de[, "Pr(>|z|)"], fdr.level = 0.05 )$qvalues # control for false disovery rate
de <- de[order( de$qvalue, decreasing = FALSE ), ] # order by global statistical evidence
knitr::kable(head( de ))
```

|  | Estimate | Pr(>|z|) | qvalue |
| --- | --- | --- | --- |
| IGHM\_ENSG00000211899 | -5.486870 | 1.0e-07 | 0.0005661 |
| IGHV4-34\_ENSG00000211956 | -5.486870 | 1.0e-07 | 0.0005661 |
| NA\_ENSG00000251301 | -5.032614 | 1.0e-07 | 0.0005661 |
| RASSF4\_ENSG00000107551 | -3.439349 | 1.1e-06 | 0.0024541 |
| CD86\_ENSG00000114013 | -3.664782 | 8.0e-07 | 0.0024541 |
| DNAJA1P3\_ENSG00000215007 | -3.439349 | 1.1e-06 | 0.0024541 |

In *scater* the `plotExpression` function enables the convenient visualisation of expression values for a set of features. Here, the expression values for the six most DE genes for expression frequency between patients are shown. The units for expression in the plot can be defined with the `exprs_values` argument (the expression values must exist with the provided name in the `assayData` slot of the SCESet object; if not the default `exprs` values will be used, with a warning). As with other plots in *scater* we can use phenotype data variables to define the colour and shapr of the points.

```
plotExpression(scDataQC, rownames(de)[1:6], x = "sample_type", ncol = 3)
```

```
plotExpression(scDataQC, rownames(de)[1:6], ncol = 3, exprs_values = "counts",
               x = "sample_type", colour_by = "log10_counts_endogenous_features")
```

```
plotExpression(scDataQC, rownames(de)[1:6], exprs_values = "norm_exprs", 
               x = "sample_type", colour_by = "total_features", 
               shape_by = "c1_machine", ncol = 3)
```

One natural question is if differentially expressed genes are co-expressed. Clustering of the genes, using Gaussian mixture models, suggests that the immunoglobulin genes with increased expression in patient B form a tight coexpression cluster (shown in red) that is distinct from the other differentially expressed genes.

```
library( "mclust" )

coexprsA <- cor( t( norm_exprs(scDataQC)[row.names(de)[de$qvalue < 0.05], 
                              sm == "cell from patient A"] ),  # rank coexpression
                 method = "spearman" )
coexprsA <- data.frame( cmdscale( (1 - coexprsA) / 2 ) ) # multidimensional scaling of the coexpression
clustA <- Mclust( coexprsA, modelNames = "VVV" ) # model based clustering of the differentially expressed genes

coexprsB <- cor( t( norm_exprs(scDataQC)[row.names(de)[de$qvalue < 0.05], 
                                         sm == "cell from patient B"] ),  # repeat
                method = "spearman" )
coexprsB <- data.frame( cmdscale( (1 - coexprsB) / 2 ) ) 
clustB <- Mclust( coexprsB, modelNames = "VVV" ) 

gns <- fData( scDataQC )[row.names( de )[de$qvalue < 0.05], "hgnc_symbol"] # differentially expressed genes' symbols
cls <- c( "firebrick", "grey30" )[clustB$classification] # colour by clustering
eff <- rank( de$Estimate[de$qvalue < 0.05] ) / 60 # size by relative expression in patient B 

par( mfrow = c( 2, 1 ) )
plot( clustA, what = "density", bty = "n", xlab = "patient A dimension one",  ylab = "patient A dimension two" )
text( coexprsA[, 1], coexprsA[, 2], gns, cex = eff, col = cls )
plot( clustB, what = "density", bty = "n", xlab = "patient B dimension one",  ylab = "patient B dimension two" )
text( coexprsB[, 1], coexprsB[, 2], gns, cex = eff, col = cls )
```

---

## 7. SESSION INFORMATION

Scater has been tested on Mac OS X and Linux environments and requires the R packages:

- Biobase
- BiocGenerics
- ggplot2
- methods

and imports the packages:

- biomaRt
- data.table
- dplyr
- edgeR
- grid
- limma
- matrixStats
- plyr
- reshape2
- rhdf5
- rjson
- viridis

This case study was run using the following platform and R package versions:

```
## R version 3.3.1 (2016-06-21)
## Platform: x86_64-apple-darwin13.4.0 (64-bit)
## Running under: OS X 10.11.6 (El Capitan)
## 
## locale:
## [1] en_GB.UTF-8/en_GB.UTF-8/en_GB.UTF-8/C/en_GB.UTF-8/en_GB.UTF-8
## 
## attached base packages:
## [1] stats4    parallel  stats     graphics  grDevices utils     datasets 
## [8] methods   base     
## 
## other attached packages:
##  [1] qvalue_2.5.2                BiocStyle_2.1.24           
##  [3] RUVSeq_1.7.2                edgeR_3.15.2               
##  [5] limma_3.29.20               EDASeq_2.7.2               
##  [7] ShortRead_1.31.1            GenomicAlignments_1.9.6    
##  [9] SummarizedExperiment_1.3.82 Rsamtools_1.25.1           
## [11] GenomicRanges_1.25.93       GenomeInfoDb_1.9.7         
## [13] Biostrings_2.41.4           XVector_0.13.7             
## [15] IRanges_2.7.14              S4Vectors_0.11.13          
## [17] scran_1.1.9                 BiocParallel_1.7.8         
## [19] EBImage_4.15.4              scater_1.1.13              
## [21] ggplot2_2.1.0               Biobase_2.33.3             
## [23] BiocGenerics_0.19.2        
## 
## loaded via a namespace (and not attached):
##   [1] Hmisc_3.17-4            aroma.light_3.3.1      
##   [3] RcppEigen_0.3.2.9.0     igraph_1.0.1           
##   [5] plyr_1.8.4              lazyeval_0.2.0         
##   [7] sp_1.2-3                shinydashboard_0.5.1   
##   [9] splines_3.3.1           digest_0.6.10          
##  [11] htmltools_0.3.5         viridis_0.3.4          
##  [13] tiff_0.1-5              magrittr_1.5           
##  [15] cluster_2.0.4           annotate_1.51.0        
##  [17] matrixStats_0.50.2      R.utils_2.3.0          
##  [19] xts_0.9-7               jpeg_0.1-8             
##  [21] colorspace_1.2-6        rrcov_1.3-11           
##  [23] dplyr_0.5.0             RCurl_1.95-4.8         
##  [25] tximport_1.1.3          genefilter_1.55.2      
##  [27] lme4_1.1-12             survival_2.39-5        
##  [29] zoo_1.7-13              gtable_0.2.0           
##  [31] zlibbioc_1.19.0         MatrixModels_0.4-1     
##  [33] kernlab_0.9-24          car_2.1-3              
##  [35] prabclus_2.2-6          DEoptimR_1.0-6         
##  [37] abind_1.4-5             SparseM_1.7            
##  [39] VIM_4.5.0               scales_0.4.0           
##  [41] sgeostat_1.0-27         DESeq_1.25.0           
##  [43] mvtnorm_1.0-5           DBI_0.5                
##  [45] GGally_1.2.0            sROC_0.1-2             
##  [47] Rcpp_0.12.6             xtable_1.8-2           
##  [49] laeken_0.4.6            proxy_0.4-16           
##  [51] foreign_0.8-66          mclust_5.2             
##  [53] Formula_1.2-1           vcd_1.4-1              
##  [55] FNN_1.1                 RColorBrewer_1.1-2     
##  [57] fpc_2.1-10              acepack_1.3-3.3        
##  [59] modeltools_0.2-21       flexmix_2.3-13         
##  [61] reshape_0.8.5           XML_3.98-1.4           
##  [63] R.methodsS3_1.7.1       nnet_7.3-12            
##  [65] locfit_1.5-9.1          dynamicTreeCut_1.63-1  
##  [67] labeling_0.3            reshape2_1.4.1         
##  [69] AnnotationDbi_1.35.4    munsell_0.4.3          
##  [71] tools_3.3.1             RSQLite_1.0.0          
##  [73] pls_2.5-0               evaluate_0.9           
##  [75] stringr_1.1.0           cvTools_0.3.2          
##  [77] fftwtools_0.9-7         yaml_2.1.13            
##  [79] knitr_1.14              robustbase_0.92-6      
##  [81] nlme_3.1-128            mime_0.5               
##  [83] quantreg_5.26           formatR_1.4            
##  [85] R.oo_1.20.0             biomaRt_2.29.2         
##  [87] pbkrtest_0.4-6          beeswarm_0.2.3         
##  [89] png_0.1-7               e1071_1.6-7            
##  [91] smoother_1.1            tibble_1.2             
##  [93] statmod_1.4.26          robCompositions_2.0.2  
##  [95] geneplotter_1.51.0      pcaPP_1.9-60           
##  [97] stringi_1.1.1           highr_0.6              
##  [99] GenomicFeatures_1.25.16 trimcluster_0.1-2      
## [101] lattice_0.20-33         Matrix_1.2-7.1         
## [103] nloptr_1.0.4            lmtest_0.9-34          
## [105] data.table_1.9.6        cowplot_0.6.2          
## [107] bitops_1.0-6            httpuv_1.3.3           
## [109] rtracklayer_1.33.12     R6_2.1.3               
## [111] latticeExtra_0.6-28     hwriter_1.3.2          
## [113] gridExtra_2.2.1         vipor_0.4.3            
## [115] boot_1.3-18             MASS_7.3-45            
## [117] assertthat_0.1          destiny_1.99.3         
## [119] chron_2.3-47            rhdf5_2.17.5           
## [121] rjson_0.2.15            diptest_0.75-7         
## [123] mgcv_1.8-14             rpart_4.1-10           
## [125] grid_3.3.1              class_7.3-14           
## [127] minqa_1.2.4             rmarkdown_1.0          
## [129] mvoutlier_2.0.6         TTR_0.23-1             
## [131] scatterplot3d_0.3-37    shiny_0.13.2           
## [133] ggbeeswarm_0.5.0
```

```
## Figures for the paper
if ( requireNamespace("cowplot") ) {
    ## QC figure
    scData$well_type <- "single cell"
    scData$well_type[scData$sample_type == "blank (empty site) control"] <-
        "blank control"
    scData$well_type[scData$sample_type == "bulk control from patient A"] <-
        "bulk control"
    scData$well_type[scData$sample_type == "bulk control from patient B"] <-
        "bulk control"
    p1 <- plot( scData, colour_by = "well_type", exprs_values = "counts") +
        theme(legend.position = c(0.8, 0.25))
    p2 <- plotPCA( scData, shape_by = "sample_type", 
                   pca_data_input = "pdata", detect_outliers = TRUE,
                   selected_variables = c("cdna_recovered_in_ng_per_ul_no_miss",
                                          "pct_counts_top_100_features",
                                          "total_features", 
                                          "pct_counts_feature_controls",
                                          "n_detected_feature_controls", 
                                          "log10_counts_endogenous_features",
                                          "log10_counts_feature_controls"))
    p3 <- plotQC( scData[fData( scData )$use, pData( scData )$use], 
        type = "highest-expression", feature_names_to_plot = "gene_symbol",
        col_by_variable = "sample_type", n = 20) + coord_cartesian(xlim = c(0, 6)) +
        theme(axis.text.y = element_text(size = 10),
              axis.title = element_text(size = 11))
    p4 <- plotQC(scData, type = "exprs")
    p5 <- plotQC( scData[fData( scData )$use, scData$use], 
        type = "explanatory-variables", 
        variables = c("pct_counts_top_100_features", "total_features", 
                      "c1_machine",
                      "n_detected_feature_controls", 
                      "log10_counts_endogenous_features",
                      "sample_type") ) + 
        xlab(expression(paste(R ^ 2, " (% variance explained; log10-scale)")))
    p6 <- plotQC( scData[fData( scData )$use, scData$use], 
        type = "find-pcs", variable = "c1_machine")
    figplot1 <- cowplot::plot_grid(p1, p2, p3, p4, p5, p6, 
                                  labels = letters, ncol = 3,
                                  label_size = 20)
    cowplot::save_plot("../figures/figure3.pdf", figplot1, ncol = 3, nrow = 2,
                       base_height = 3.5, base_aspect_ratio = 1.4)
    cowplot::save_plot("../figures/figure3.png", figplot1, ncol = 3, nrow = 2,
                       base_height = 3.5, base_aspect_ratio = 1.4)
    cowplot::save_plot("../figures/figure3.jpg", figplot1, ncol = 3, nrow = 2,
                       base_height = 3.5, base_aspect_ratio = 1.4, dpi = 350)

    ## alternative version for better readability
    figplot1_alt <- cowplot::ggdraw() +
        cowplot::draw_plot(p1, 0.01, .5, 0.28, .48) +
        cowplot::draw_plot_label(letters[1], x = 0, y = 1, size = 20) +
        cowplot::draw_plot(p2, 0.29, .5, 0.35, .48) +
        cowplot::draw_plot_label(letters[2], x = 0.29, y = 1, size = 20) +
        cowplot::draw_plot(p4, 0.64, .5, 0.36, .48) +
        cowplot::draw_plot_label(letters[3], x = 0.63, y = 1, size = 20) +
        cowplot::draw_plot(p3, 0.01, 0, 0.5, .48) +
        cowplot::draw_plot_label(letters[4], x = 0, y = 0.49, size = 20) +
        cowplot::draw_plot(p5, 0.52, 0, .48, .48) +
        cowplot::draw_plot_label(letters[5], x = 0.515, y = 0.49, size = 20)
    
    cowplot::save_plot("../figures/figure3_alt.pdf", figplot1_alt, ncol = 3, nrow = 2,
                       base_height = 3.2, base_aspect_ratio = 1.4)
    cowplot::save_plot("../figures/figure3_alt.png", figplot1_alt, ncol = 3, nrow = 2,
                       base_height = 3.2, base_aspect_ratio = 1.4)
    cowplot::save_plot("../figures/figure3_alt.jpg", figplot1_alt, ncol = 3, nrow = 2,
                       base_height = 3.2, base_aspect_ratio = 1.4, dpi = 350)
    
    ## save find-pcs plot to add to supplement
    cowplot::save_plot("../figures/find-pcs_c1_machine.pdf", p6,
                       base_height = 5, base_aspect_ratio = 1.1)
    cowplot::save_plot("../figures/find-pcs_c1_machine.png", p6,
                       base_height = 5, base_aspect_ratio = 1.1, dpi = 350)
    cowplot::save_plot("../figures/find-pcs_c1_machine.jpg", p6,
                       base_height = 5, base_aspect_ratio = 1.1, dpi = 350)
    
    ## dimension reduction figure
    ww <- which(featureNames(scDataQC) %in% rownames(de)[c(1:2, 5)])
    # ww <- which(featureNames(scDataQC) %in% c("IGHM_ENSG00000211899", 
    #                                           "IGHV4-34_ENSG00000211956",
    #                                           "CD86_ENSG00000114013"))
    # ww <- which(featureNames(scDataQC) %in% c("IGHM", 
    #                                           "IGHV4-34",
    #                                           "CD86"))
    featureNames(scDataQC)[ww] <- as.character(fData(scDataQC)$hgnc_symbol[ww])
    feats_to_plot <- as.character(fData(scDataQC)$hgnc_symbol[ww])
    featureNames(scData)[most_exprs_ccycle[7]] <- 
        fData(scData)$hgnc_symbol[most_exprs_ccycle[7]]

    p1 <- plotPCA(scData[, scData$use], 
                  colour_by = "sample_type", theme_size = 14,
                  shape_by = "c1_machine") + ggtitle("PCA - all genes") + 
        ylab("Component 2") +
        theme(legend.position = "none")
    p2 <- plotTSNE(scData[, scData$use], 
                   colour_by = "sample_type", theme_size = 14,
                   shape_by = "c1_machine", rand_seed = 20151225) + 
        ggtitle("t-SNE - all genes") + 
        theme(legend.position = "none")
    p3 <- plotDiffusionMap( scData[, scData$use], 
                            colour_by = "sample_type",  rand_seed = 20160814,
                            shape_by = "c1_machine", theme_size = 14) + 
        ggtitle("Diffusion map - all genes") +
        theme(legend.position = "right")
    p4 <- plotPCA(scData[, scData$use], feature_set = cell_cycle_genes, 
                  colour_by = featureNames(scData)[most_exprs_ccycle[7]], 
                  theme_size = 14) + 
        ggtitle("PCA - cell cycle genes") + ylab("Component 2") +
        #geom_point(aes(colour = colour_by, shape = shape_by), alpha = 0.6, size = 4) +
        theme(legend.position = "none")
    p5 <- plotTSNE(scData[, scData$use], feature_set = cell_cycle_genes, 
                   colour_by = featureNames(scData)[most_exprs_ccycle[7]], 
                   rand_seed = 20160814, theme_size = 14) + 
        #geom_point(aes(colour = colour_by, shape = shape_by), alpha = 0.6, size = 4) +
        ggtitle("t-SNE - cell cycle genes") + 
        theme(legend.position = "none")
    p6 <- plotDiffusionMap( scData[, scData$use], feature_set = cell_cycle_genes, 
                            colour_by = featureNames(scData)[most_exprs_ccycle[7]], 
                            theme_size = 14, rand_seed = 20160814) + 
        #geom_point(aes(colour = colour_by, shape = shape_by), alpha = 0.6, size = 4) +
        ggtitle("Diffusion map - cell cycle genes") +
        theme(legend.position = "right")
    
    plt_exprs <- plotExpression(
        scDataQC, feats_to_plot, exprs_values = "norm_exprs", 
        x = "sample_type", colour_by = "total_features", ncol = 3, size = 3,
        theme_size = 14) + ylab("Expression") + xlab(NULL)
    
    figplot2 <- cowplot::ggdraw() +
        cowplot::draw_plot(p1, 0, .65, 0.29, .35) +
        cowplot::draw_plot_label(letters[1], x = 0, y = 1, size = 24) +
        cowplot::draw_plot(p2, 0.3, .65, 0.29, .35) +
        cowplot::draw_plot_label(letters[2], x = 0.3, y = 1, size = 24) +
        cowplot::draw_plot(p3, 0.59, .65, 0.42, .35) +
        cowplot::draw_plot_label(letters[3], x = 0.6, y = 1, size = 24) +
        cowplot::draw_plot(p4, 0, .3, 0.29, .35) +
        cowplot::draw_plot_label(letters[4], x = 0, y = 0.65, size = 24) +
        cowplot::draw_plot(p5, 0.3, .3, 0.29, .35) +
        cowplot::draw_plot_label(letters[5], x = 0.29, y = 0.65, size = 24) +
        cowplot::draw_plot(p6, 0.59, .3, 0.37, .35) +
        cowplot::draw_plot_label(letters[6], x = 0.59, y = 0.65, size = 24) +
        cowplot::draw_plot(plt_exprs, 0.005, 0, 0.99, 0.29) +
        cowplot::draw_plot_label(letters[7],  x = 0, y = 0.29, size = 24)
    figplot2
    
    cowplot::save_plot("../figures/figure4.pdf", figplot2, nrow = 3, ncol = 3,
                       base_height = 2.7, base_aspect_ratio = 2)
    cowplot::save_plot("../figures/figure4.png", figplot2,  ncol = 3, nrow = 3,
                       base_height = 2.7, base_aspect_ratio = 2)
    cowplot::save_plot("../figures/figure4.jpg", figplot2,  ncol = 3, nrow = 3,
                       base_height = 2.7, base_aspect_ratio = 2, dpi = 350)
    
    if ( requireNamespace("RUVSeq")) {
        library( "RUVSeq" )
        scDataRUV <- filter(scData, use)
        scDataRUV <- scDataRUV[fData(scData)$use | fData(scData)$is_feature_control,]
        scIdx <- matrix(-1, ncol = max(table(scDataRUV$sample_type)), nrow = 2)
        scIdx[1,] <- which(scDataRUV$sample_type == "cell from patient B")
        tmp <- which(scDataRUV$sample_type == "cell from patient A")
        scIdx[2, 1:length(tmp)] <- tmp
        cIdx <- featureNames(scDataRUV)
        ## RUVs
        ruvs <- RUVs(counts(scDataRUV), cIdx, k = 1, scIdx = scIdx, isLog = FALSE)
        norm_counts(scDataRUV) <- ruvs$normalizedCounts
        exprs(scDataRUV) <- edgeR::cpm.default(norm_counts(scDataRUV), log = TRUE, 
                                               prior.count = scDataRUV@logExprsOffset)
        plt_norm_ruvs <- plotPCA(
            scDataRUV, exprs_values = "exprs", shape_by = "c1_machine", 
            colour_by = "sample_type", size_by = "total_features", 
            scale_features = TRUE) + 
            ggtitle(paste("PCA - RUVs normalisation: k =", 1)) +
            theme(legend.position = "right")

        plt_pca_endog_norm <- plt_pca_endog_norm + 
            theme(legend.position = "none")

        plt_sf_norm_resid <- plt_sf_norm_resid + 
            theme(legend.position = "none")
    

        figplot3 <- cowplot::plot_grid(plt_pca_endog_norm, plt_sf_norm_resid,
                                       plt_norm_ruvs,
                                       labels = letters, ncol = 3,
                                       rel_widths = c(0.75, 0.75, 1), label_size = 18)
        figplot3

        cowplot::save_plot("../figures/figure5.pdf", figplot3, ncol = 3, nrow = 1,
                           base_height = 2.7, base_aspect_ratio = 1.5)
        cowplot::save_plot("../figures/figure5.png", figplot3, ncol = 3, nrow = 1,
                           base_height = 2.7, base_aspect_ratio = 1.5)
        cowplot::save_plot("../figures/figure5.jpg", figplot3, ncol = 3, nrow = 1,
                           base_height = 2.7, base_aspect_ratio = 1.5, dpi = 350)

    
    }
    
    
}
```

```
## Warning in `levels<-`(`*tmp*`, value = if (nl == nL) as.character(labels)
## else paste0(labels, : duplicated levels in factors are deprecated

## Warning in `levels<-`(`*tmp*`, value = if (nl == nL) as.character(labels)
## else paste0(labels, : duplicated levels in factors are deprecated

## Warning in `levels<-`(`*tmp*`, value = if (nl == nL) as.character(labels)
## else paste0(labels, : duplicated levels in factors are deprecated
```

```
## `stat_bindot()` using `bins = 30`. Pick better value with `binwidth`.
```

```
## Warning in `levels<-`(`*tmp*`, value = if (nl == nL) as.character(labels)
## else paste0(labels, : duplicated levels in factors are deprecated

## Warning in `levels<-`(`*tmp*`, value = if (nl == nL) as.character(labels)
## else paste0(labels, : duplicated levels in factors are deprecated
```

```
## `stat_bindot()` using `bins = 30`. Pick better value with `binwidth`.
## `stat_bindot()` using `bins = 30`. Pick better value with `binwidth`.
## `stat_bindot()` using `bins = 30`. Pick better value with `binwidth`.
```
