# Supplementary material for: Scater: pre-processing, quality control, normalization and visualization of single-cell RNA-seq data in R
Source: Bioinformatics. 2017 Jan 14;33(8):1179–86. doi: 10.1093/bioinformatics/btw777 (PMC5408845; doi:10.1093/bioinformatics/btw777)
Supplement: Supplementary Data [file btw777_supp.zip › supplement_figs.pdf]

Supplementary Figures—“*scater:* pre-processing, quality control, normalisation and visualisation of single-cell RNA-seq data in R”

Davis J. McCarthy [1,2,5*], Kieran R. Campbell [2,4], Aaron T. L. Lun [6] and Quin F. Wills [2,3]
[1] *European Molecular Biology Laboratory - European Bioinformatics Institute (EMBL-EBI), Hinxton CB10 1SD, United Kingdom;*
[2] *Wellcome Trust Centre for Human Genetics, University of Oxford, Roosevelt Drive, Oxford OX3 7BN, United Kingdom;*
[3] *Weatherall Institute for Molecular Medicine, University of Oxford, John Radcliffe Hospital, Oxford OX3 9DS, United Kingdom;*
[4] *Department of Physiology, Anatomy and Genetics, University of Oxford, South Parks Road, Oxford OX1 3QX, United Kingdom;*
[5] *St Vincent's Institute of Medical Research, 41 Victoria Parade, Fitzroy Victoria 3065, Australia; and*
[6] *CRUK Cambridge Institute, University of Cambridge, Robinson Way, Cambridge CB2 0RE, United Kingdom.*

Supplementary material and figures—details of package dependencies; an overview of the SCESet class; an overview of the *scater* ecosystem; examples of using the *scater* GUI.

Contact: davis@ebi.ac.uk

Details of package dependencies

The package builds on many other R packages: *Biobase* and *BiocGenerics* for core Bioconductor functionality [13]; *plyr* [20], *reshape2* [19], *dplyr* [22], *data.table* [8] and *magrittr* [3] for reading and tidying data; *ggplot2* [21] for plotting; *biomaRt* [9] for feature annotation; *edgeR* [16] for computation of normalisation size factors and counts-per-million values; *limma* [15] for efficient fitting of linear models to features; *rhdf5* [11], *rjson* [7] and *tximport* [17] for reading in transcript-level expression values; *viridis* [12] for perceptually-uniform colour maps for plotting; *parallel* for parallel computation; *matrixStats* [4] for computation of summary statistics from matrices; *cowplot* [24] for attractive plotting themes; *destiny* [2] for producing diffusion maps; *Rtsne* [14] for producing t-SNE plots; *mvoutlier* [10] for multivariate outlier detection from PCA of QC metrics; *roxygen2* [23], *BiocStyle* [13], *knitr* [25] and *rmarkdown* [1] for generating documentation; and *testthat* [18] for unit testing. As well as functioning in the usual R environments, *scater* also has a GUI built using *shiny* [6] and *shinydashboard* [5] for intuitive and interactive data visualisation. Calling the `scater_gui` function from within an R session opens up the GUI in a web browser.

Entry points for third-party tools from *scater*

The *scater* package serves to prepare data for a wide variety of downstream analyses with third-party tools. Given the diverse nature of analyses that can be done with scRNA-seq data, the entry points for various third-party tools in terms of data preparation and transformation/format can vary. Below we discuss entry points from *scater* into example third-party tools representing major categories of downstream analysis.

a. Differential expression analysis with scde *or* edgeR Differential expression analysis tools for RNA-seq data, including *scde* and *edgeR* take raw counts as input data and can handle known batch effects in their statistical models. Therefore, the upstream data processing with *scater* is straightforward: QC on cells and genes should be carried out with *scater* as usual, and the count data supplied to the DE tool. Accessing the count matrix is as simple as applying the counts() function to an SCESet object. For an *edgeR* analysis, size-factors computed with *scran* should also be supplied, and the convertTo function from *scran* makes it very easy to convert an SCESet object from a *scater* workflow to a DGEList object needed for an *edgeR* analysis.

b. Clustering with SC3 Clustering results can be negatively impacted by the inclusion of poor quality cells, so QC of cells and genes as usual with *scater* is necessary before supplying data to a clustering algorithm. In general, clustering algorithms do not account internally for batch effects and similar, so it will often be desirable to normalise expression data with size factors and regress out known batch effects or other technical effects. In the case of clustering with *SC3*, a QC'd and normalised SCESet object can be supplied directly to the sc3 function for clustering.

c. Pseudotime estimation with monocle As with clustering, current pseudotime estimation methods do not internally account for batch effects. Thus, before applying pseudotime tools, data should be QC'd with *scater* to remove problematic cells and genes. Size-factor normalisation of data is advisable, and the normaliseExprs function in *scater* can be used to regress out known batch or technical effects. Once a filtered and normalised SCESet object has been

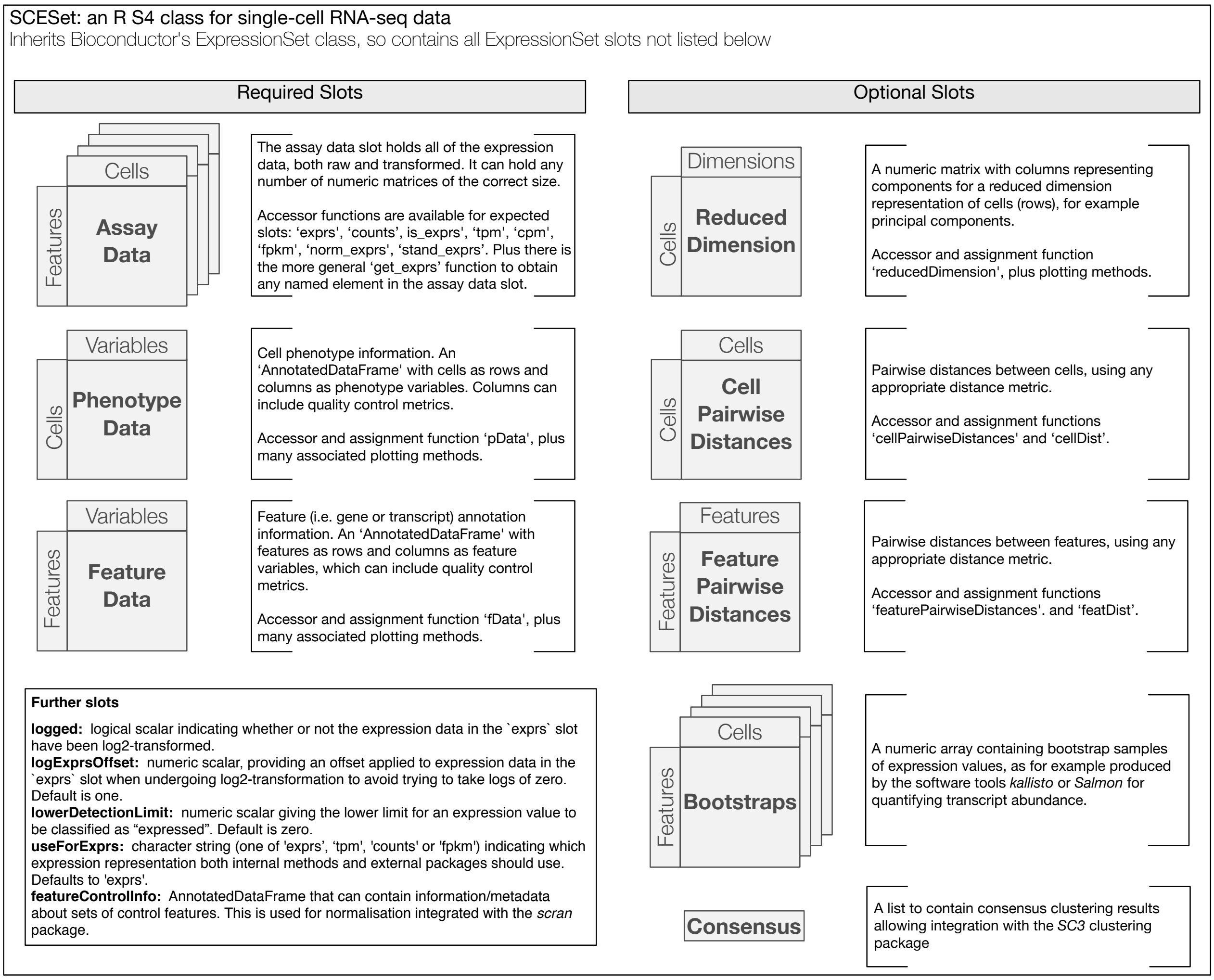

FIG. 1: An overview of the SCESet class that underpins the *scater* package. Building on Bioconductor's ExpressionSet class, it is a fully-featured, sophisticated and flexible data class tailored to scRNA-seq data.

obtained, the convertTo function in *scran* can be used to convert the SCESet object to a CellDataSet object used in *monocle*.

The three examples above demonstrate that the QC steps in *scater* are necessary before any downstream analyses. The entry point from *scater* varies for different third-party tools, but is nevertheless straightforward in most cases.

Supplementary Figures

[1] Allaire, J. J., Cheng, J., Xie, Y., McPherson, J., Chang, W., Allen, J., Wickham, H., Atkins, A., and Hyndman, R. (2016). rmarkdown: Dynamic Documents for R.
[2] Angerer, P., Haghverdi, L., Büttner, M., Theis, F. J., Marr, C., and Buettner, F. (2015). destiny: diffusion maps for large-scale single-cell data in R. *Bioinformatics*.
[3] Bache, S. M. and Wickham, H. (2014). Magrittr: A forward-pipe operator for R. *R package version*.
[4] Bengtsson, H. (2016). matrixStats: Functions that Apply to Rows and Columns of Matrices (and to Vectors).
[5] Chang, W. (2015). shinydashboard: Create Dashboards with 'Shiny'.
[6] Chang, W., Cheng, J., Allaire, J. J., Xie, Y., and McPherson, J. (2016). shiny: Web Application Framework for R.
[7] Couture-Beil, A. (2014). rjson: JSON for R.

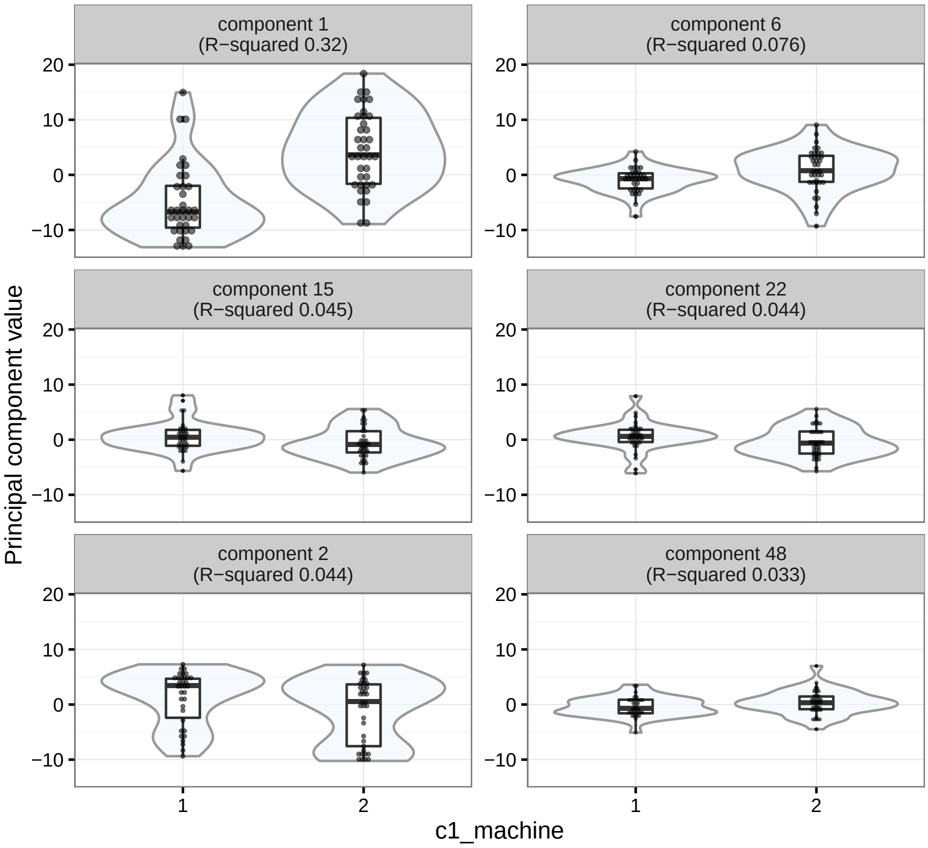

FIG. 2: A QC plot produced by the plotQC function in *scater* showing violin, scatter- and boxplots of principal component values against the C1 machine used for each cell for the six principal components most strongly correlated with C1 machine used.

[8] Dowle, M., Srinivasan, A., Short, T., and Lianoglou, S. (2015). data.table: Extension of Data.frame.

[9] Durinck, S., Moreau, Y., Kasprzyk, A., Davis, S., De Moor, B., Brazma, A., and Huber, W. (2005). BioMart and Bioconductor: a powerful link between biological databases and microarray data analysis. *Bioinformatics*, **21**(16), 3439–3440.

[10] Filzmoser, P. and Gschwandtner, M. (2015). mvoutlier: Multivariate outlier detection based on robust methods.

[11] Fischer, B. and Pau, G. (2016). rhdf5: HDF5 interface to R.

[12] Garnier, S. (2016). viridis: Default Color Maps from 'matplotlib'.

[13] Huber, W., Carey, V. J., Gentleman, R., Anders, S., Carlson, M., Carvalho, B. S., Bravo, H. C., Davis, S., Gatto, L., Girke, T., Gottardo, R., Hahne, F., Hansen, K. D., Irizarry, R. A., Lawrence, M., Love, M. I., MacDonald, J., Obenchain, V., Oleś, A. K., Pagès, H., Reyes, A., Shannon, P., Smyth, G. K., Tenenbaum, D., Waldron, L., and Morgan, M. (2015). Orchestrating high-throughput genomic analysis with Bioconductor. *Nature methods*, **12**(2), 115–121.

[14] Krijthe, J. (2015). Rtsne: T-Distributed Stochastic Neighbor Embedding using Barnes-Hut Implementation. *0.10, URL http://CRAN. R-project. org/package= Rtsne*.

[15] Ritchie, M. E., Phipson, B., Wu, D., Hu, Y., Law, C. W., Shi, W., and Smyth, G. K. (2015). limma powers differential expression analyses for RNA-sequencing and microarray studies. *Nucleic acids research*, **43**(7), e47.

[16] Robinson, M. D., McCarthy, D. J., and Smyth, G. K. (2010). edgeR: a Bioconductor package for differential expression analysis of digital gene expression data. *Bioinformatics*, **26**(1), 139–140.

[17] Soneson, C., Love, M. I., and Robinson, M. D. (2015). Differential analyses for RNA-seq: transcript-level estimates improve gene-level inferences. *F1000Research*, **4**, 1521.

[18] Wickham, H. (2011). testthat: Get started with testing. *The R journal*, **3**(1), 5–10.

[19] Wickham, H. (2012). reshape2: Flexibly reshape data: a reboot of the reshape package. *R package version*.

[20] Wickham, H. (2015). plyr: Tools for splitting, applying and combining data. R package version 1.8. 1. *R Found. Stat. Comput., Vienna*.

[21] Wickham, H. (2016). *ggplot2: Elegant Graphics for Data Analysis*. Springer.

[22] Wickham, H. and Francois, R. (2015). dplyr: A grammar of data manipulation. *R package version 0. 4*, **1**, 20.

[23] Wickham, H., Danenberg, P., and Eugster, M. (2015). roxygen2: In-Source Documentation for R.

[24] Wilke, C. O. (2016). cowplot: Streamlined Plot Theme and Plot Annotations for 'ggplot2'.

[25] Xie, Y. (2013). *Dynamic Documents with R and knitr*, volume 29. CRC Press.

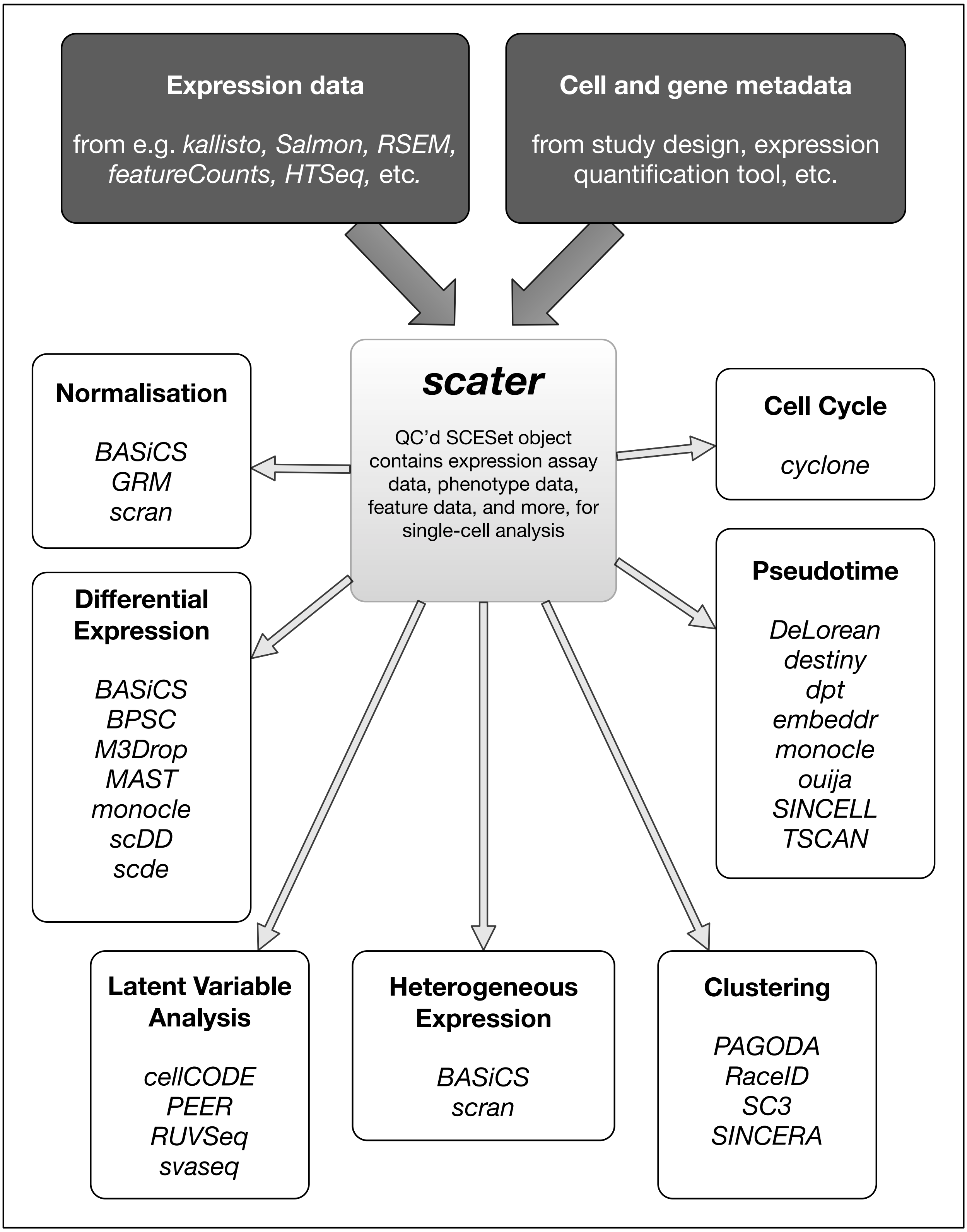

FIG. 3: An overview of the *scater* ecosystem. The SCESet class in *scater* acts as a convenient hub for datasets so that many other methods and tools implemented in R can be applied.

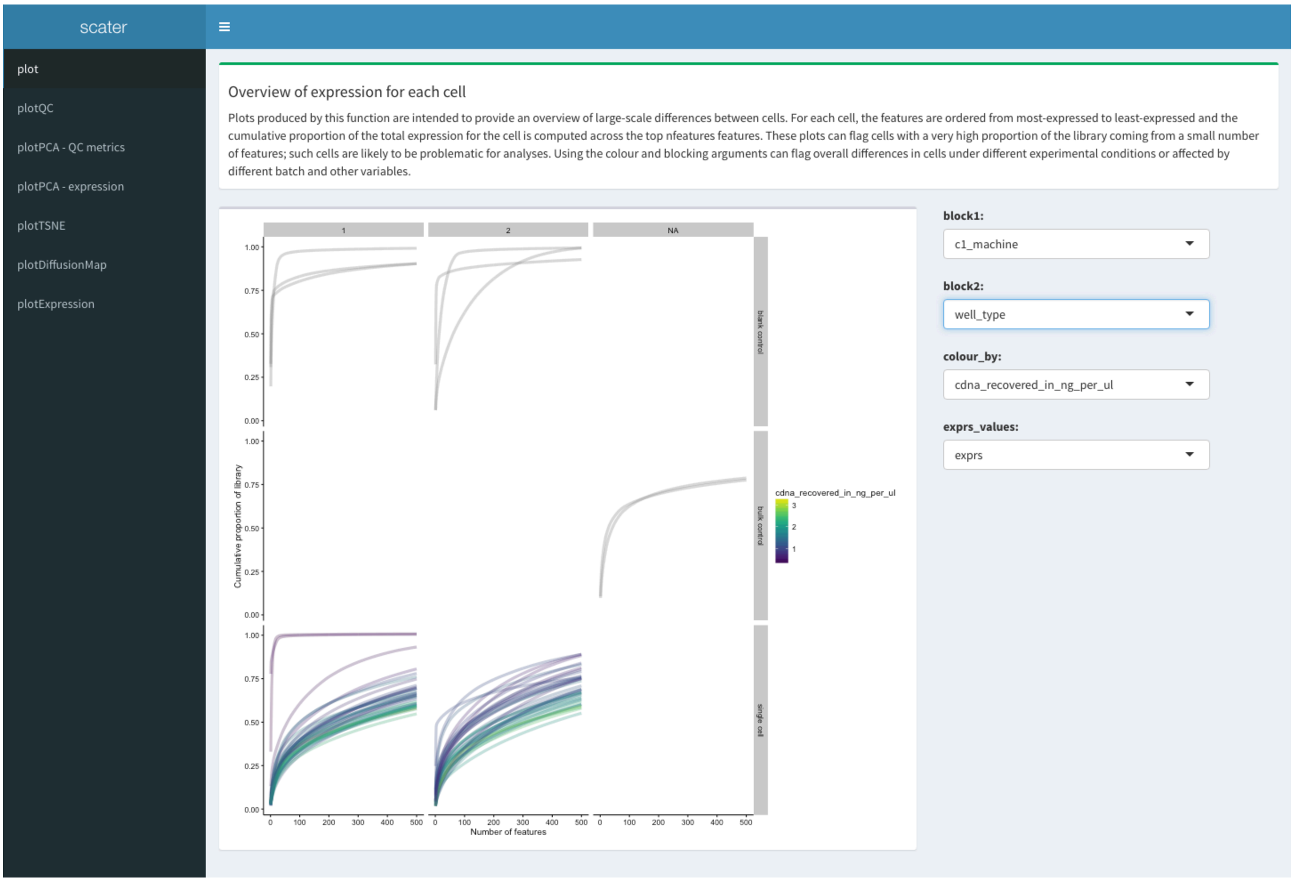

FIG. 4: The landing page for the *scater* graphical user interface (GUI).

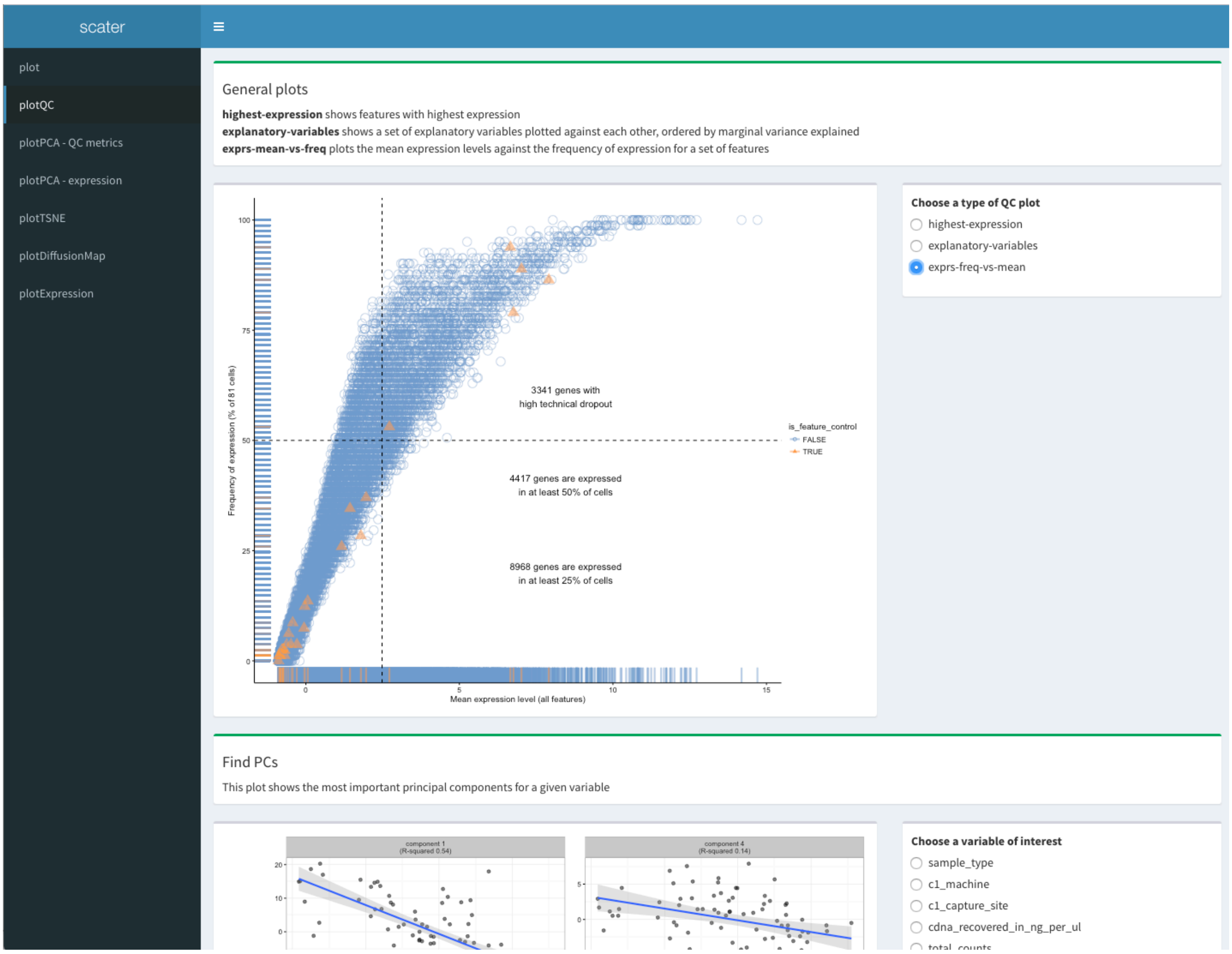

FIG. 5: The `plotQC` page for the *scater* graphical user interface (GUI).

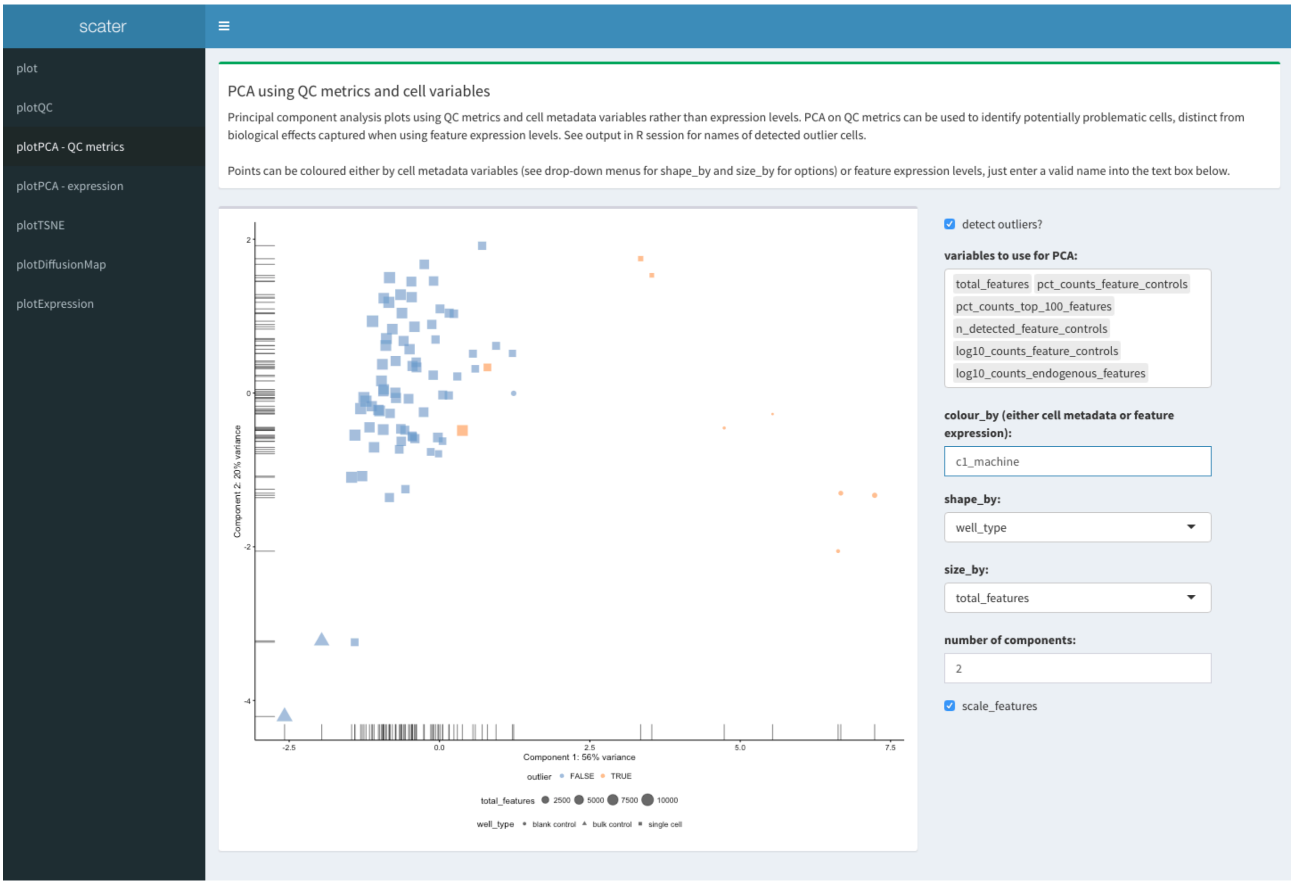

FIG. 6: The `plotPCA - QC` page for the *scater* graphical user interface (GUI).
